# From Universal Health Coverage services packages to budget appropriation: the long journey to implementation

**DOI:** 10.1136/bmjgh-2022-010755

**Published:** 2023-05-14

**Authors:** Agnès Soucat, Ajay Tandon, Eduardo Gonzales Pier

**Affiliations:** 1 Health & Social Protection, Agence Francaise de Developpement, Paris, France; 2 World Bank Group, Washington, District of Columbia, USA; 3 Palladium Group Inc, Washington, District of Columbia, USA

**Keywords:** Health policies and all other topics, Health economics, Health policy, Health systems, Health services research

## Abstract

Essential packages of health services (EPHS) potentially contribute to universal health coverage (UHC) financing through several pathways. Generally, expectations on what an EPHS can achieve for health financing are high, yet stakeholders rarely spell out mechanisms to reach desired outcomes. This paper analyses how EPHS relate to the three health financing functions (revenue raising, risk pooling and purchasing) and to public financial management (PFM). Our review of country experiences found that using EPHS to directly leverage funds for health has rarely been effective. Indirectly, EPHS can translate into increased revenue through fiscal measures, including health taxes. Through improved dialogue with public finance authorities, health policy-makers can use EPHS or health benefit packages to communicate the value of additional public spending connected with UHC indicators. Overall, however, empirical evidence on EPHS contribution to resource mobilisation is still pending. EPHS development exercises have been more successful in advancing resource pooling across different schemes: EPHS can help comparing performance of coverage schemes, occasionally leading to harmonisation of UHC interventions and identifying gaps between health financing and service delivery. EPHS development and iterative revisions play an essential role in core strategic purchasing activities as countries develop their health technology assessment capacity. Ultimately, packages need to translate into adequate public financing appropriations through country health programme design, ensuring funding flows directly address obstacles to increased coverage.

**SUMMARY BOX**

- Essential packages of health services (EPHS) for universal health coverage (UHC) are often aspirational, with great hopes for an increase in public resources, yet are usually disconnected from the revenue-raising question.
- EPHS can help to progress towards harmonisation of UHC interventions and provide the basis for pooling funds.
- Package definitions and iterative revisions play an essential role in core strategic purchasing activities leading countries to develop their capacity in health technology assessment.
- Ultimately, packages need to translate into country programme design and implementation leading to adequate public financing appropriations.

## Introduction

 Over the past three decades, many countries have invested in essential packages of health services (EPHS) development, aiming at the progressive realisation of universal health coverage (UHC). The focus on both UHC objectives of service coverage and financial risk protection (FRP) implies both that how much a country spends on health and the way a health system is financed are important. High-performing health financing systems for UHC are those where public funding levels are predictable, prepaid funds are pooled across population groups sharing the financial risk of ill health, and spending delivers service coverage and FRP for all people.^1^

## Background

Defining an explicit publicly financed EPHS is a foundational element of UHC reforms across countries.^2 3^ Countries have typically used technical approaches based on burden of disease and cost-effectiveness criteria, sometimes adding considerations such as acceptability, feasibility, equity and budgetary impact.^4^ Some countries including Tunisia and Thailand used participatory approaches and societal dialogue.^5 6^ Financing schemes for EPHS are diverse. In Armenia, EPHS has taken a ‘pyramid’ shape with the poor getting a larger publicly financed package and the well-off co-paying complementary private health insurance.^7^ In Indonesia, contributions are collected from the well-off in exchange for better hoteling amenities and greater provider choice.^8^ In Malaysia, Sri Lanka and Brazil, publicly financed EPHS are universal, although the well-off self-select to use private care.^9^

**Figure 1:**
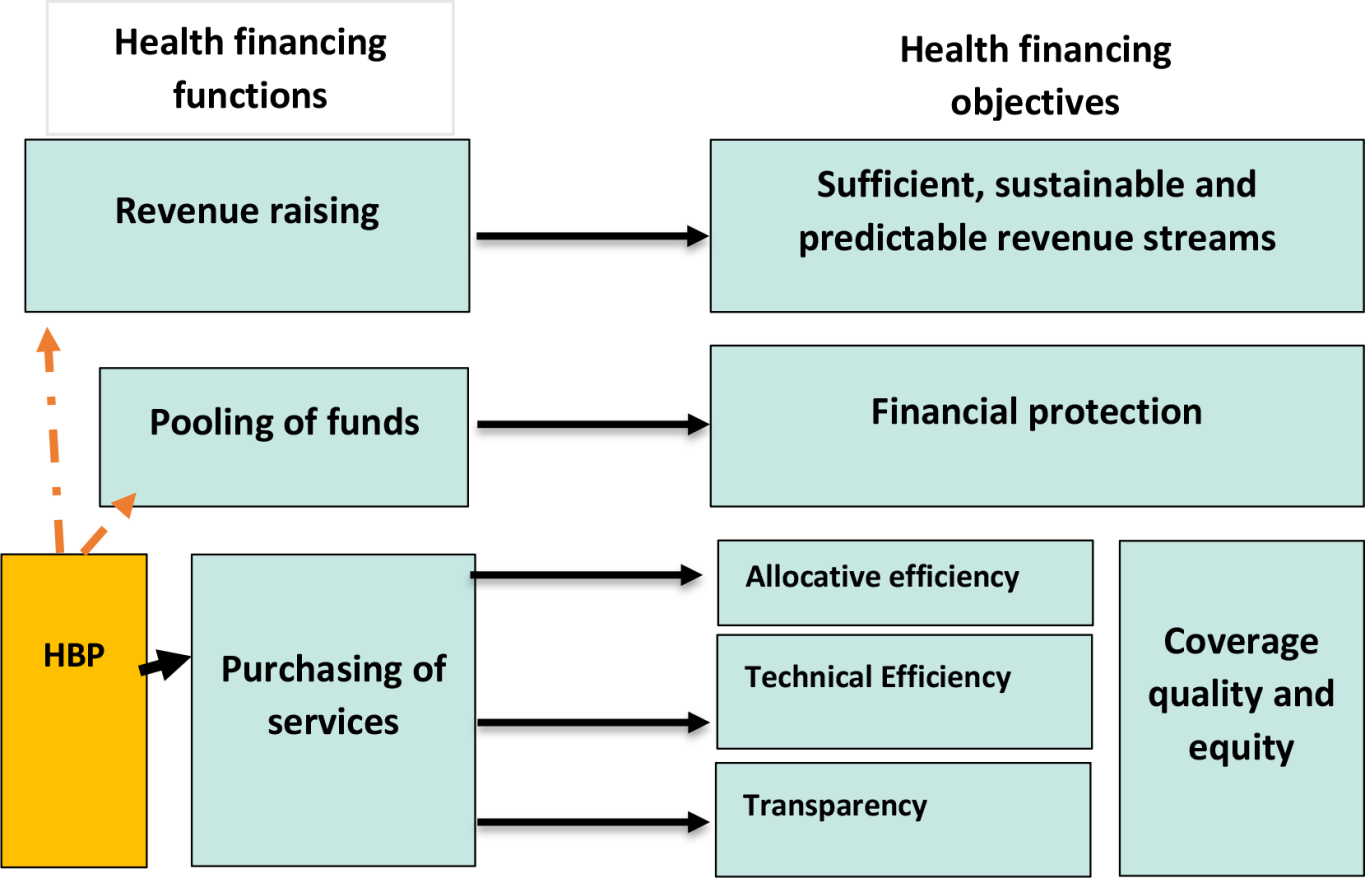
The link between EPHS and health finance policy objectives. EPHS, essential packages of health services.

Clearly defining which services should benefit from public financing is central to achieving UHC. This paper analyses how EPHS relate to the three health financing functions (revenue raising, risk pooling and purchasing)^10^ and to PFM. The EPHS design process, costing,^11^ priority setting and implementation are directly associated with the subfunction of strategic purchasing, indirectly reinforcing pooling (through coverage alignment on breadth and depth) but less directly linked to revenue generation. Nevertheless, the process of developing an EPHS contributes to all health financing core functions (figure 1).

## Revenue raising: the ‘fiscal space’ question

In most low-income and middle-income countries (LMICs) that have undertaken an EPHS exercise, the total cost of the proposed package invariably exceeds public financing available. Despite great hopes, few of these costing exercises have translated into additional resources suggesting that this exercise—while useful for health policy-makers in identifying priorities—is not the most effective instrument in making the case for additional public resources for UHC. Even when the EPHS were deemed affordable, resources rarely materialised. Ghana, for example, developed a well-designed EPHS, but there is no evidence that it translated into increased budgets.^12^ Package development in Kenya did not foster increase in health resources.^13^ A review of six countries—Eswatini, Ethiopia, Malawi, Nigeria, Rwanda and South Africa—shows a systemic disconnect between EPHS processes and health financing policies and frameworks.^14^

This series of papers reviewed the recent experience from Afghanistan, Ethiopia, Pakistan, Somalia, Sudan and Zanzibar-Tanzania in setting their EPHS. Box 1 shows a summary of the EPHS’ role in the health financing dialogue for the six countries included in this series.

Box 1 **Selected experience in the health financing dialogue in six countries**

This series of papers reviewed the recent experience of Afghanistan, Ethiopia, Pakistan, Somalia, Sudan and Zanzibar-Tanzania in setting their own essential packages of health services (EPHS). We find that none of these countries conducted a financing dialogue as part of the package discussions. All teams stopped at identifying a theoretical financing gap and estimating its size, comparing a theoretical ‘fiscal space’ to the cost of the package on a per capita basis. This ‘gap analysis’ approach is often referred to in the EPHS literature as the ‘financing’ step, with no evidence provided as to the effectiveness of this simple analysis in improving the health financing framework of a given country. These six countries did not see appreciable changes in domestically sourced public financing for health, for example, in Ethiopia and Afghanistan, these expenditures have remained largely stagnant in real per capita terms over 2015–2019, and even if other countries where these have increased, the growth rates have been lower than those among comparator countries. (figure A) In Pakistan, the EPHS development process informed the design of a donor funded joint programme. EPHS development aimed at raising externally financed resources to complement some increases in domestic financing for health, although overall numbers remain low and uncertain. Overall, none of the six countries undertook a dialogue on how to raise revenue, create or consolidated entitlement or pooling mechanism, identify system inefficiencies to address, establish a new payment provider mechanism or defining a specific programme to be included in the country multiyear budget (figure A).

**Figure A:**
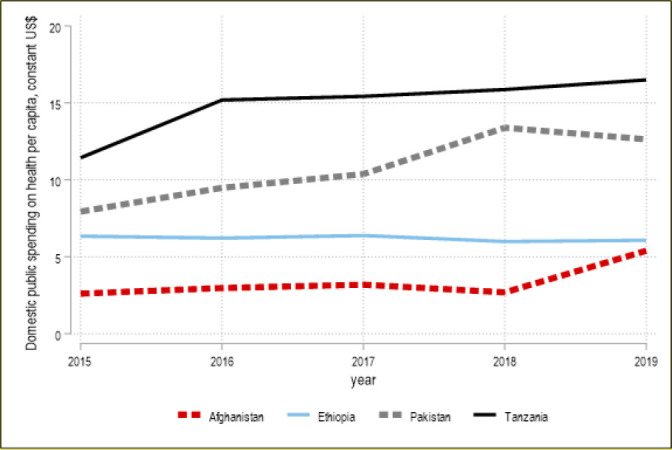
Evolution of public spending 2015–2019 in four countries.

**Figure 2:**
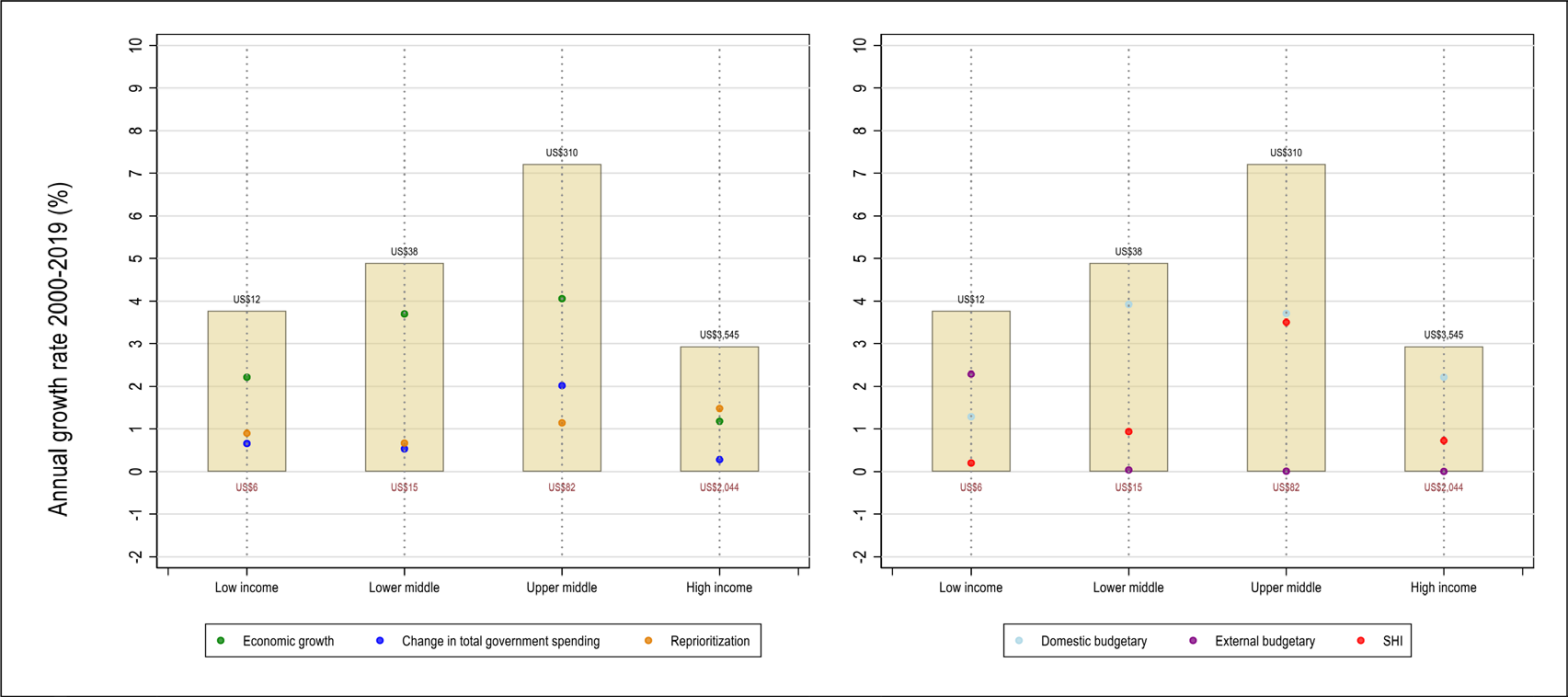
Drivers of public financing for health by macrofiscal determinants (left panel) and by fiscal source (right panel), 2000–2019. SHI, social health insurance.

Fiscal space, the potential to increase public spending, is not sector specific. It depends on macroeconomic growth and stability, how effective governments are at raising revenues, debt sustainability, the capacity to manage overall expenditures, and—in lower-income settings—on external financing. Public spending on health is, in turn, determined by earmarked revenues (such as payroll taxes for social health insurance (SHI) or health taxes) and the relative importance assigned to the health sector in the general and subnational budget appropriation processes. Historically, public spending on health has grown mostly through the impetus of economic growth and improvements in government revenue efforts.^15^ Few countries have increased public financing through dramatic reprioritisation of sectoral allocations to health. From 2000 to 2019, public financing for health in low-income countries doubled in real terms from US$6 per capita in 2000 to US$12 in 2019, a 3.8% average real growth per year. For lower-middle-income countries, it more than doubled during the same period from US$15 to US$38, a 4.9% average per year increase. For upper-middle-income countries, it more than tripled in real terms from US$82 in 2000 to US$310 in 2019 or 7.2% per year (figure 2).

**Figure 3:**
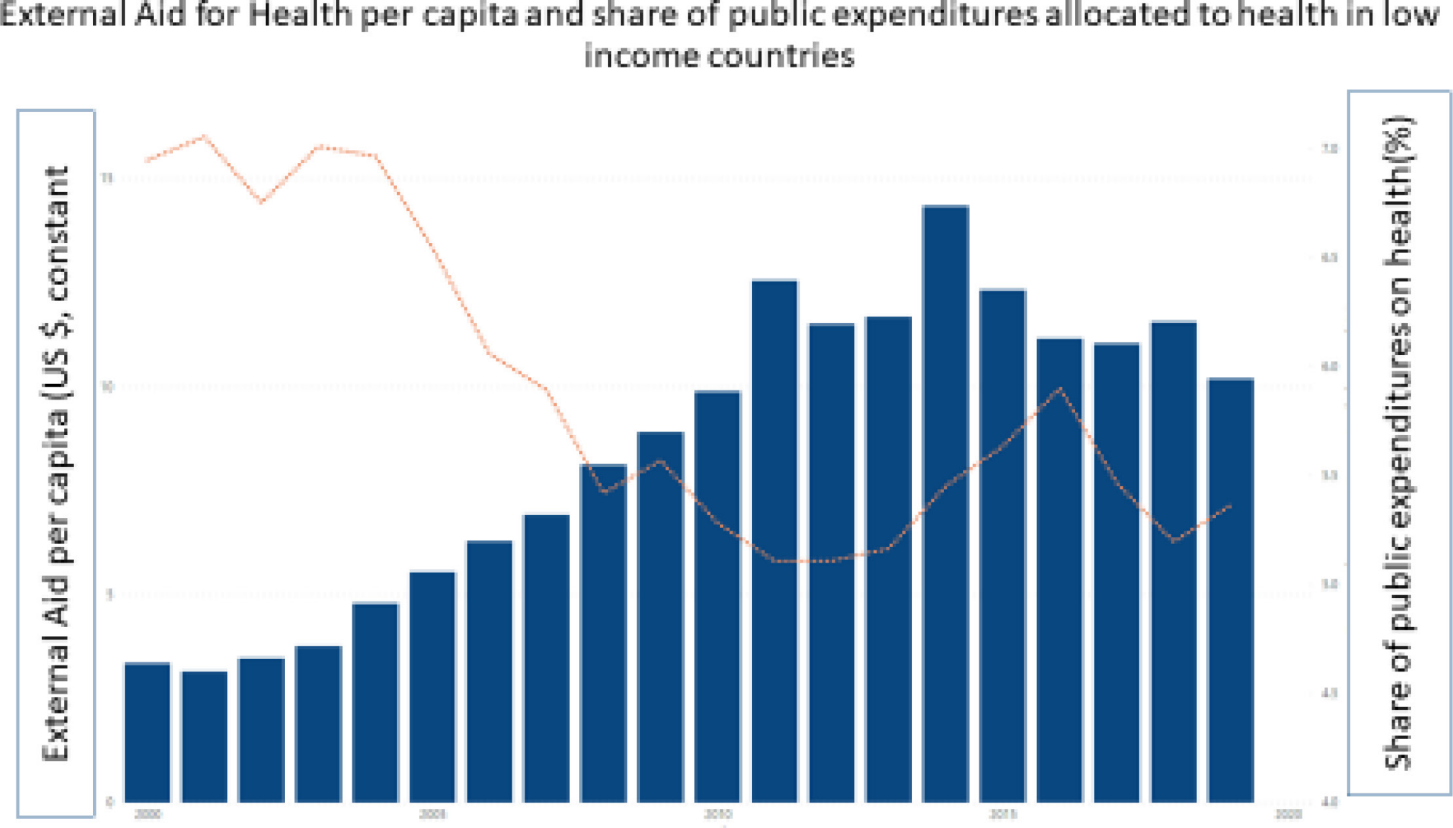
Crowding out government spending on health from domestic sources in aid-dependent countries (external health aid and government domestic health spending), 2005–2019.

This growth in public spending for health can be broken down into three components: economic growth, changes in government spending as a share of Gross Domestic Product, and changes in health’s priority in government budgets. Economic growth—the increase in government revenues due to increased size of the economy—has been the largest contributor to growth in public spending for health across LMICs, followed by overall increases in general spending. Increased priority for health has played a relatively smaller role. In low-income countries, reprioritisation can be explained by higher external funding channelled via government budgets allocations. In fact, figure 3 shows how external sources mostly substituted for domestic funding that declined in aid-dependent countries between 2000 and 2019.^16^ Most low-income, aid-dependent countries have not developed UHC financing frameworks setting them on a sustainable trajectory by 2030 (figure 3).

Where a conducive macrofiscal environment is not enough, greater prioritisation of health is often difficult to realise. First, governments face competing demands and other sectors also present well-articulated claims to public funding. Second, many countries do not fully execute health budgets, weakening their case for increased funding. Policy-makers may perceive the health sector as inefficient, lacking a clear strategy to optimise available resources and improve service coverage. Finally, in some countries, identifying a ‘funding gap’ based on costing of an EPHS may lead to calls for more ‘innovative finance’^17^ at both national and global level, making the case for more private finance which can widen inequalities. Sometimes, the inertial increases in public financing for health derived from favourable macrofiscal environments masked relatively low priority for health in government budgets.

Advocates of expanded service benefit packages often argue based on theoretical economic returns related to improved health outcomes such as lives saved. This argument is not the most convincing for decision-makers when health outcomes overall, and averted premature mortality in particular are often the products of multisectoral action.^18^ For example, country evidence shows a very clear relationship between female education or access to electricity and child mortality.^19^ While the health sector acknowledges other sectors’ health contributions, it is less adept at promoting how investments in health also contribute to other sectors (eg, poverty alleviation, educational attainment and labour productivity), thereby leveraging shared desirable societal benefits to improve budget share.

We think a more convincing argument would be to connect increases in public financing for EPHS with improvements in UHC indicators (service coverage and FRP), as these are more easily attributable to government reform efforts within the health sector. The cost of not implementing reforms should be clearly spelled out, in terms of lost health benefits and lower associated labour productivity. Low levels of public financing for health can contribute to inefficiency; for example, primary healthcare facilities without health workers or without adequate diagnostics and medicines make wasteful investments in infrastructure.

One clear pathway for benefit packages to increase revenue is the inclusion of behavioural interventions supported by tax policy. These taxes or subsidies are designed to change the relative prices of commodities and services to promote healthier consumption patterns. Health taxes on alcohol, tobacco and sugar-sweetened beverages (SSB) have been implemented with success in many countries.^20^ Removing subsidies or taxation on fossil fuels can be considered health taxes, as they reduce the burden of disease related to air pollution. These taxes have potential to raise health budgets. Earmarking is typically not desirable, but it can help increase public support for fiscal reform. In France, for example, the tax on SSB is earmarked to the agriculture worker health scheme.^21^ Additional funding amounts may be significant in some countries (eg, Philippines, Mexico). However, there is evidence (eg, from Ghana and Kazakhstan) that over time, there are compensating reductions in allocations from more discretionary revenues.^22^

Introducing local and regional common goods for health into EPHS (eg, vector control, epidemiological surveillance, public health messaging and other population-based services) can constitute a strong argument for additional financing. One of the strongest arguments to justify adequate levels of public finance is that common goods are by definition underfunded if left to market forces and can only be financed through general taxation.^23^ This issue is particularly salient since the COVID-19 pandemic, and the high cost of inaction is apparent to all finance ministries and treasuries worldwide.^24^

Designing EPHS may also improve advocacy for resource mobilisation by creating a narrative of progress and success on outcomes, as happened in Mexico.^25^ The capacity of Ministry of Finance officials, members of congress and other budget decision makers regarding the health budget improves when dialogue is conducted in terms of health conditions and interventions—and expected health outputs. Without explicit EPHS, the narrative goes back to staffing, payroll, health commodities and services, and other budget line items, which have a lower potential to resonate with citizens and policy-makers. To be credible, the narrative should also build the perception of the sectors’ effectiveness and efficiency to use the funds well and deliver results.

The potential of using the EPHS to increase revenue for health is limited. The disconnect between aspirational health plans and available financial and other resources is identified as the most common failing of existing benefits plans in low-income countries, leading to implicit rationing that is especially harmful for the poor.^26^ To be credible, health services packages should be elaborated within the boundaries of likely available fiscal space. Given uncertainties about what will become available as well as the cost, existing inefficiencies and levels of service use, it is often preferable to not overly specify the package and rather incorporate flexibility and adjustment capacity. The Oregon Health Insurance Experiment in the USA started with a tentative fiscal envelope and defined the EPHS within available resources.^27^

## Pooling resources: from vertical streams to comprehensive package funding

Pooling is the key health financing function that allows cross-subsidisation from rich to poor and from healthy to sick. From a health financing perspective, a ‘benefit package’ consists of the services (and conditions of access to them) that the purchaser(s) will pay for from pooled funds. The benefit package definition and implementation process is a critical tool establishing entitlements for a group of people covered by a health financing pool, supporting a unified framework. A unified benefit framework enables mapping to funding sources, facilitating efforts to either pool them together (eg, combining general revenue transfers and social insurance contributions into one pool) or to make them explicitly complementary. It can show how population and cost coverage for those services can be mapped to different funding sources (both on the supply side, ‘insurance/purchaser side’, and unfunded parts for which copayments apply), enabling depiction of which parts are covered from different pools.

This is then an enabler for explicit complementarity among funding sources. Such a framework exists in France (‘Mutuelles’ and Kyrgyzstan (contributory SHI) . In both cases, there is a ‘main pool’, which funds a benefit package for the entire population, together with copayments, and then there is (a) complementary insurance pool(s) for some or all of the copayments.^28^ Defining an EPHS can help define this unified national benefits framework for the entire population on the basis of scientific evidence as done in the UK NICE (National Institute for Care Excellence) and France (High Authority for Health), on the basis of health technology assessment (HTA).

A specific challenge is to blend supply and demand-side financing. Indonesia’s SHI scheme Jaminan Kesehatan Nasional (JKN)^29^ which covers about three-fourths of the population, accounts for only one-quarter of total health financing. The remainder comes from budgetary line-item public financing and user fees to public providers and out-of-pocket fees to private providers. This ‘partial reimbursement’ SHI model—common in other countries such as India, Philippines, Vietnam^30^—is on the other hand not adequately leveraged to be truly complementary to other revenue. In the same way, Thailand’s public providers have their salaries paid through supply side budgets while the National Health Security Office (NHSO) pays for outputs under the UC Scheme.

The UHC package costing can identify potential efficiency gains through service integration and comprehensive planning. In contexts where funding is highly fragmented, with multiple public health programmes and sources of funding, designing and costing an EPHS creates a level playing field to assess funding needs, harmonising wage levels, incentives and service delivery assumptions. An EPHS will identify economies of scope to be achieved by pooling resources. Identifying an explicit UHC package consolidates evidence from various service providers and public health programmes within a common service delivery framework. This helps reduce overhead levels and better accounts for common costs like human resources and infrastructure plus reduces overlap. Reducing inefficiencies generates budget space for improved access to other interventions.

Recently, EPHS have mainstreamed externally funded vertical programmes into financing schemes funding broader set of health services. For example, Nigeria, Kenya, Vietnam and Tanzania successfully integrated their family planning and HIV/AIDS treatment programmes into UHC schemes while transitioning away from external funding.^31 32^

Health services packages may also be an effective tool to standardise access criteria across fragmented health insurance schemes. An EPHS can also help identify gross inequalities in how governments subsidise different groups through different pools. Defining a national service package establishes a standard to compare current expenditures by different schemes. Assessing EPHS has flagged unfair access built into segregated insurance pools financed from general taxation. A study conducted in Thailand in 1997 showed how different schemes (low income, voluntary, social security and civil service medical benefit schemes) received vastly disparate per capita subsidies for different service packages, highlighting the need to develop an equalised package for all.^33^ Eventually, this led to merging most of the schemes with an increasingly harmonised subsidy. Explicit mapping of a UHC package to multiple funding pools allow to pay providers on the basis of marginal cost: Thai public providers have their salaries paid through supply side budgets while the NHSO pays for outputs under the UHC scheme.^34^

EPHS can clearly estimate required resources to be transferred to a scheme in charge of paying providers and defining how to best target subsidies. An open-ended package leads to implicit rationing and inequities as realised benefits are a function of service provider capacity, where richer areas have greater response capacity and the better off are more capable at advocating for preferential access. In Indonesia, for example, JKN has an open-ended benefit package. All medically necessary services are covered under the scheme with no co-payments, no caps and no limits (other than a list of services not covered like plastic surgery and fertility treatments). Because of the open-ended package and an important part of provider payment (inpatient part) rewarding the volume of inpatient care, and also because of major supply side imbalances across the country, the national pool results in reverse cross-subsidisation with financing allocations for poorer and rural areas under the single payer arrangement subsidising richer and urban areas. Current reforms aim to define a minimum explicit package of servicesa UHC package like under Chile’s Universal Access with Explicit Guarantees (AUGE) programmea UHC package to ensure equitable and quality access to benefits.

## Strategic purchasing: health services packages to maximise efficiency and equity

Making the purchasing of healthcare strategic is probably the most important outcome of a well-designed package. Clarifying which services are to be purchased as a priority—allocative efficiency—is often seen as the main role of designing a package. The design and costing of the health services package can even be considered a subcore function of strategic purchasing.^35^

Investment in national institutions is a key area that could provide evidence for policy-makers to steer strategic purchasing including benefit package design and adjustment. While many discrete country exercises have been conducted, few led to capacity building or the creation of an agency mandated with HTA or strategic purchasing.^36^ A key element of institutional construction is to build such an agency and integrate a three-dimensional process—data analysis, dialogue and decision—whether at national or at regional level.^37^ Lebanon, Tunisia and Morocco have started building such institutions.^38^

EPHS can help countries improve value for money and maximise the health benefits per financing unit, freeing up resources by identifying cost saving interventions. Defining a package may also improve technical efficiency. First, a clear EPHS can improve collaboration between public and private delivery systems by leveraging public financing to purchase benefit packages from private providers. Second, even with explicitly defined benefits, it is not just how providers are paid (fee for service, capitation, diagnostic related groups), but also how much. Inadequate levels of public financing for explicitly and poorly costed benefits are a recipe for misalignment between promises and reality. Reforms targeted towards the poor can become poor programmes because of inadequate finance. When reforms are implemented and partially funded, but the EPHS is not explicitly spelled out, and/or these reforms are not adequately costed and financed, this spells trouble. The perception of reforms on paper, coupled with implicit rationing and skewed incomplete results, starts a new cycle of problems. A costed package can be the basis for calculating provider payments, as in the early days of UHC in Thailand.^39^ Third, with a growing burden of chronic conditions, package definitions help to emphasise integrated financing across levels of care.

## From fiscal space to health budget space

**Figure 4:**
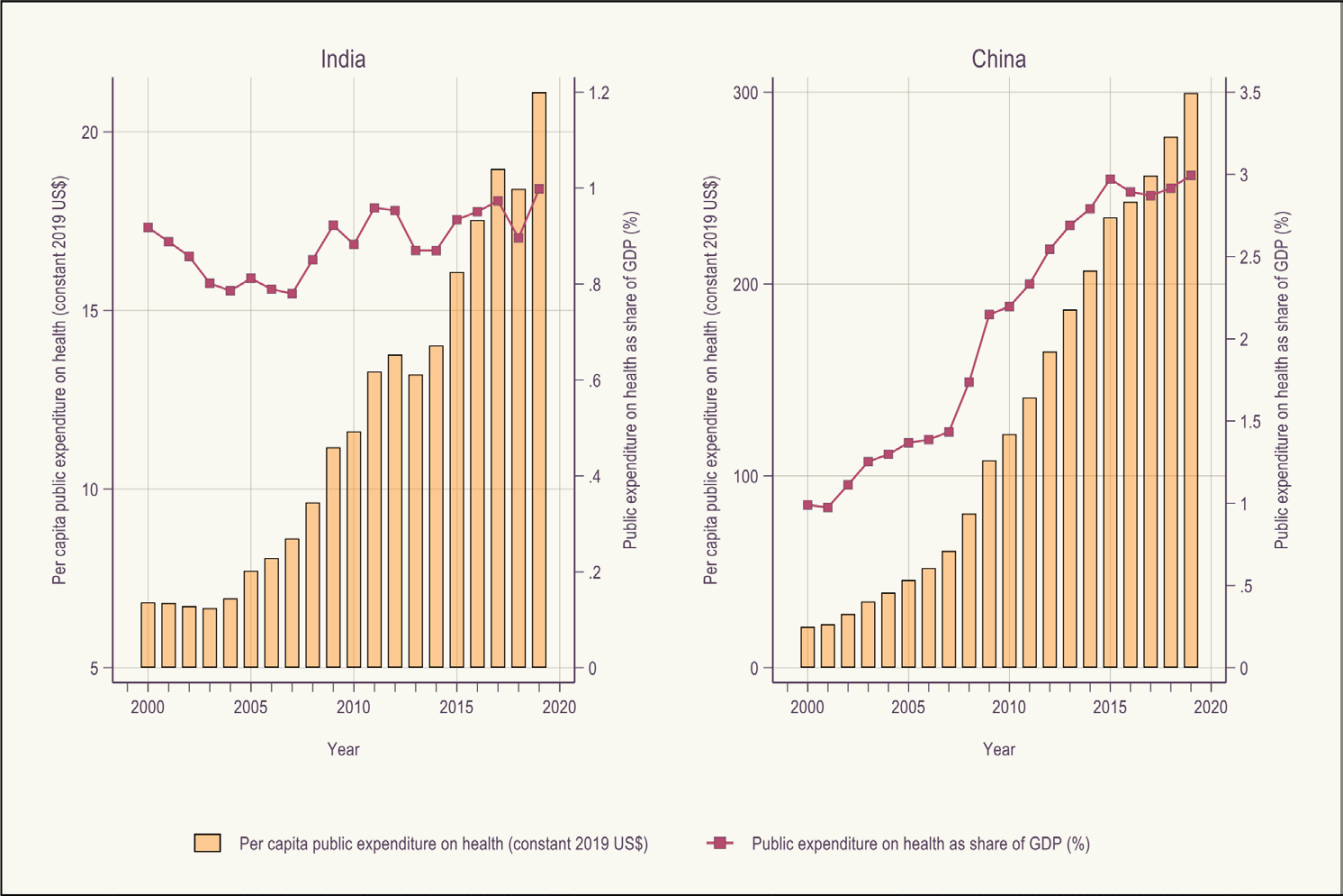
Public expenditure in health for India and China 2000–2019.

In countries with conducive macrofiscal environments, EPHS exercises can be financed even if the government health budget share has remained unchanged. In such settings, it is key to be ready to deploy additional health resources. Comparing India’s experience to that of China highlights this point. Figure 4 shows public spending on health in constant 2019 dollars and as a share of GDP for 2000–2019.

In 2000, public spending on health was roughly 1% of GDP in both countries, amounting to US$7 in India and US$21 in China. Although public spending on health in India remained at 1% of GDP over 20 years, it tripled in real per capita levels s reaching US$21 in 2019, solely due to dramatic GDP growth. New resources enabled a series of reforms, beginning with the rural primary healthcare expansion (the 2005 National Rural Health Mission expanded reproductive, maternal, newborn, adolescent and child health). There was no increase in priority of health in the government budget over this period. China, by contrast, saw public financing for health increase almost fifteen times over 2000–2019, from a combination of economic growth, increasing government revenue efforts and higher priority for health in the government budget. Public spending on health in China stands now at 3.5% of GDP, one of the largest fiscal space for health growths seen in the past two decades. But China is also a cautionary tale. Part of expenditure growth was very likely the product of largely unmanaged fee-for-service reimbursement, as well as retaining percentage copayments (coinsurance), so there was no improvement in financial protection over this period despite the shift in the relative proportion of public and private spending.^40^

Feasibility is a central concern. A feasible set of UHC EPHS is a set of services that can be feasibly financed under existing country circumstances.^41^ Increasing public resources requires identifying strategies to unlock bottlenecks service packages uptake. To be useful, the EPHS must be further translated into a broader programme definition as part of the budget cycle. Modalities to fund transfers and provider payments should be defined, as well as expected results in terms of direct benefits to population (access and use).

Linking resources to intended results through programme budgeting is a means to translate EPHS from theory to practice, enabling both alignment of budgets to the promised services and enhancing budget execution.^42^ A key dimension of this is loosening of the rigidity of line item budgeting.^43^ Ultimately, budgets are to better match the EPHS and shift from input-based to output-based payment.

Examples of such programmes are conditional intergovernmental grants, performance-based financing programmes, capitation transfers, health insurance programmes, including for specific groups and investment programmes (table 1).

**Table 1:** Theory of change: how health services packages might generate financing for UHC

Health financing core function	Pathway and country examples
Resource mobilisation	Capacity to advocate for health taxes (South Africa, Mexico, Philippines, Morocco) Identification of public/common goods for health (Iran) Improved dialogue with public finance authorities (Mexico, Pakistan)
Pooling	Coverage alignment on breadth and depth across funds (India) Equalisation of public subsidy between groups (Thailand) Addressing funding gaps of programmes (Indonesia) Targeting the poor for inclusion to the same coverage scheme (South Africa, India) Explicit complementarity of different revenue sources for the package (Kyrgyzstan) Introduction of marginal cost insurance programmes, combining supply side financing from the budget (eg, for salaries) with output-based payment by an explicit purchasing agency (Thailand Universal Coverage Scheme)
Strategic purchasing	Allocative efficiency through priority setting (mostly Cost Effectiveness Analysis) and establishing a HTA agency/practice (France, Lebanon, Norway, UK, Tunisia, India) Technical efficiency through costing exercises, improved collaboration between public and private sectors, integration of financing through levels of care and/or identification of provider payment mechanisms that can improve linkages between pooling and service delivery of explicit benefits (France, Thailand)

HTA, health technology assessment; UHC, universal health coverage.

Finally, public financing emanates from the demand from taxpayers and citizens for what the public purse should fund. Service packages need to integrate the broad view of citizens on how best to allocate their taxes. Where public financing is limited, it is best targeted towards the poor or for priority services. A key common element across many UHC reforms—for example, in China, Indonesia, Mexico, Philippines, Thailand and Vietnam—is the creation of a publicly subsidised programme expanding unremarkable coverage for the poor using general government revenues. Key is to move from scheme to system thinking and differentially support the poor while maintaining a universal vision.

The key distinction is how the subsidies flow to finance EPHS. This can be done directly where the budget is allocated to accredited public and private providers (through subsidies, eg, line item or global budget or case reimbursement) or indirectly when the budget instead flows to a distinct purchasing agency. India (through PMJAY for private providers—see box 2) South Africa and Mexico have direct allocation mechanisms to fund EPHS interventions while Thailand, Kyrgyzstan, Argentina, Rwanda, China, Indonesia, Ghana, each has important coverage programmes in which budget revenues flow to a distinct purchasing agencies or subnational entities to include complementary funds and then be allocated to providers.

Box 2 **The ‘Long Live India’ Ayushman Bharat programme**

Ayushman Bharat or ‘Long Live India’—the umbrella term for health sector reforms in the country—comprises two programmatic components. Roll-out of ‘health and wellness centres’ under the National Health Mission centrally sponsored scheme provide diagnostic tests, free essential medicines, and other comprehensive primary healthcare services at sub health levels. A new centrally sponsored scheme, the Pradhan Mantri Jan Aarogya Yojana, cofinanced by both the central and state governments, provides government-sponsored unremarkable health insurance coverage for a package of mostly inpatient secondary and tertiary care. The latter serves 100 million poor and near-poor families (an estimated total of 500 million individuals, roughly 40% of the country’s population) up to a maximum annual limit of ₹500 000 (~US$6750) per family that can be availed of at government and empanelled private hospitals.

Many countries—including China, Philippines and Turkey—initially expanded per case reimbursement coverage only for inpatient care, later expanding benefits to include outpatient primary and specialist care. In India, health reforms aimed at progressively realising UHC for its 1.3 billion population were initiated in 2018 (box 2).

## Conclusion

While establishing a dialogue with public finance authorities, health policy-makers should translate findings generated during EPHS development into operational approaches and programmes, to be integrated into the budget cycle. Having explicit evidence-based packages has several advantages. These include defining and implementing programme budgeting, assessing costs and whether or not promised benefits are commensurate with the overall fiscal envelope, absorbing funds that might become available due to conducive macrofiscal environments, and enabling reforms in risk pooling and strategic purchasing.

On their own, however—except where the service package might include implementation of health taxes—these advantages are not enough to facilitate realisation of additional resources from budget-holding authorities. Efforts aimed at connecting increases in public financing for EPHS with improvements in UHC indicators, identifying and removing absorption-capacity bottlenecks, mainstreaming investments in health for non-health and economic outcomes, benchmarking and demonstrating efficient and equitable improvements in attainment of health outputs are critical. These are predominantly PFM reform actions, from relaxing line item rigidities, to full move to programme budgets. Defining explicit health services packages is a useful way to enable this to happen, making links between health financing and service delivery explicit and allowing for greater results accountability.

## Data Availability

Data are available in a public, open access repository. Data are available on reasonable request.
